# Lipid and lipoprotein concentrations during pregnancy and associations with ethnicity

**DOI:** 10.1186/s12884-022-04524-2

**Published:** 2022-03-24

**Authors:** Christin W. Waage, Ibrahim Mdala, Hein Stigum, Anne Karen Jenum, Kåre I. Birkeland, Nilam Shakeel, Trond M. Michelsen, Kåre R. Richardsen, Line Sletner

**Affiliations:** 1 General Practice Research Unit (AFE), Department of General Practice, Institute of Health and Society, University of Oslo, Blindern, Post Box 1130, N-0318 Oslo, Norway; 2 Institute of Clinical Medicine, Faculty of Medicine, University of Oslo, Oslo, Norway; 3 Department of Endocrinology, Obesity and Preventive Medicine, Oslo University Hospital, Oslo, Norway; 4 Department of Obstetrics, Division of Obstetrics and Gynecology, Oslo University Hospital, Oslo, Norway; 5 Department of Physiotherapy, Oslo Metropolitan University, Oslo, Norway; 6 Department of Child and Adolescents Medicine, Akershus University Hospital, Lørenskog, Norway

**Keywords:** Ethnicity, Lipids, Pregnancy, Postpartum

## Abstract

**Background:**

To describe ethnic differences in concentrations of lipids and lipoproteins, and their changes, during pregnancy to postpartum.

**Methods:**

This was a population-based cohort study conducted in primary antenatal care in Norway. The participants (*n* = 806) were healthy, pregnant women, 59% were ethnic minorities. Outcomes were triglycerides, total cholesterol, HDL- and LDL-cholesterol, analysed from fasting blood samples drawn at gestational age (weeks) 15, 28 and 14 weeks postpartum. We performed linear regression models and linear mixed models to explore the total effect of ethnicity on the outcomes, adjusting for gestational age /week postpartum, maternal age and education. The analyses are corrected for multiple testing using the Bonferroni correction.

**Results:**

At gestational age 15, triglyceride concentrations were lower in women of African origin (1.03 mmol/mol (95% CI: 0.90, 1.16)) and higher in women of South Asian (primarily Pakistan and Sri Lanka) origin (1.42 mmol/mol (1.35, 1.49)) and East Asian (primarily Vietnam, Philippines and Thailand) origin (1.58 mmol/mol (1.43, 1.73)) compared with Western Europeans (1.26 mmol/mol (1.20, 1.32)). Women of Asian and African origin had a smaller increase in triglycerides, LDL- and total cholesterol from gestational age 15 to 28. At gestational age 28, LDL-cholesterol levels were lowest among East Asians (3.03 mmol/mol (2.72, 3.34)) compared with Western Europeans (3.62 mmol/mol (3.50, 3.74)). Triglycerides and HDL-cholesterol were lower postpartum than at gestational age 15 in all groups, but the concentration of LDL-cholesterol was higher, except in Africans. South and East Asian women had lower HDL-cholesterol and higher triglycerides postpartum, while African women had lower triglycerides than Western Europeans.

**Conclusion:**

We found significant differences in the concentrations of lipids and lipoproteins and their changes during pregnancy and the early postpartum period related to ethnic origin.

**Supplementary Information:**

The online version contains supplementary material available at 10.1186/s12884-022-04524-2.

## Background

During normal pregnancy, physiological changes in glucose and lipid metabolism occur to ensure continuous supply of nutrients to the growing foetus [1, 2]. After an initial decrease in early pregnancy, there is a progressive increase in plasma triglycerides, total cholesterol, and the lipoproteins high-density lipoprotein (HDL-) cholesterol and low-density lipoprotein (LDL-) cholesterol as pregnancy progresses [1–6]. These elevations are partly reversed postpartum, although affected by hormone levels and lactation [7–9].

The increase in maternal lipid levels has physiologic advantages. The early changes in lipid metabolism promote the accumulation of maternal fat stores in early and mid-pregnancy, allows fat mobilization as a maternal energy source in late pregnancy and facilitate transport of lipids across the placenta [1]. Triglycerides are hydrolysed by lipases on the maternal side of the syncytiotrophoblast, and free fatty acids are released and taken up by the placenta. Cholesterol is important for placental and foetal growth and maturation and necessary for steroid hormone synthesis. The foetus uses maternal cholesterol transferred across the placenta, and later in pregnancy also cholesterol from own synthesis. Cholesterol is probably delivered to the placenta by LDL-cholesterol, taken up by endocytosis. A study with data from four-vessel sampling at caesarean section indicates that HDL-cholesterol is involved in the release of cholesterol from the placenta to the foetal circulation [10].

High maternal concentrations of triglycerides, cholesterol, and LDL-cholesterol and low HDL-cholesterol are found to be associated with several pregnancy complications, such as pregnancy-related hypertension [11], pre-eclampsia [12, 13], preterm birth [14–17], gestational diabetes (GDM) [18, 19] and foetal overgrowth [20–23]. The most consistent associations seem to be present for triglycerides [2]. The prevalence of GDM, preterm birth and low birth weight differs by ethnicity, and we and others have reported ethnic differences in foetal growth [24]. Adverse lipid profiles are strongly related to cardiometabolic diseases, but of note, women with pregnancy complications like preeclampsia and GDM are also at higher risk of later cardiovascular disease (CVD) and type 2 diabetes [21, 25–27]. Ethnic differences in concentrations of lipids and lipoproteins outside pregnancy are well documented, with women of South Asian origin displaying higher triglycerides, lower HDL-cholesterol and more small, dense LDL-cholesterol particles related to insulin resistance, compared with women of Western European origin [28, 29]. Findings also suggest that women of African origin have lower triglycerides [30]. Nevertheless, few studies have explored ethnic differences in concentrations of lipids and lipoproteins in pregnancy [31–34]. Furthermore, consensus about normal reference values during pregnancy and postpartum is lacking. We hypothesized that there are ethnic differences in the concentrations of lipids and lipoproteins and their changes during pregnancy to postpartum.

## Methods

### Study population and data collection

The Stork Groruddalen study is a longitudinal, population-based cohort study of 823 healthy pregnant women (59% from non-Western ethnic minority groups) representative for the largest ethnic groups in Norway, set up in 2008 at three Child Health Clinics in Groruddalen, Oslo, Norway. The study methods have been described in detail elsewhere [35]. In short, fasting venous blood samples were drawn by trained professionals at three time points, followed by an interview the same day with few exceptions (if the women were not fasting or for logistic reasons)*.* Data from questionnaires and physical examinations including anthropometric measurements were collected according to a detailed protocol. The three study visits were scheduled to take place at gestational age (GA) (weeks) 15, at GA 28 and about four months postpartum.

Women were included in the study if they 1) lived in one of the three study districts, 2) planned to give birth at one of the two study hospitals, 3) were < 20 weeks pregnant, 4) could communicate in Norwegian, Arabic, English, Sorani, Somali, Tamil, Turkish, Urdu or Vietnamese and 5) could give informed written consent [35]. Women with known pre-pregnancy diabetes or other diseases necessitating hospital follow-up during pregnancy were excluded [35]. Some immigrants showed up later for their first antenatal visits and were therefor allowed to be included later. The minimum and maximum GA were 9 and 30 at visit 1, 20 and 33 at visit 2, and 7 and 31 at visit 3. The actual mean GA (SD) was 15.0 (3.3) at inclusion and 28.3 (1.3) at the follow-up during pregnancy, and for visit 3 the actual week postpartum was 14.2 (2.7) [35]. The interviews were performed by midwifes, and assisted by professional translators when needed.

### Primary outcomes

Fasting triglycerides, total cholesterol and HDL- and LDL-cholesterol, all measured in mmol/L at inclusion, in GA 28 and 14 weeks postpartum, were primary outcomes. Blood samples were drawn in the morning after an overnight fast and sent for routine analyses at the Akershus University Hospital and the Hormone Laboratory, Oslo University Hospital [35]. Fasting triglycerides, HDL- and total cholesterol were analysed in serum with a colorimetric method (Vitros 5.1 FS, Ortho clinical diagnostic). LDL-cholesterol was calculated using Friedewald`s formula [36] as follows: LDL-cholesterol = total cholesterol – HDL-cholesterol – (0.45 × triglycerides) mmol/L, which correlate well with directly measured LDL both early and late in pregnancy (*r* = 0.97) [37]. The analytical coefficient of variation (CVa) was 3% for triglycerides, 3.2% for HDL-cholesterol, 3.0% for total cholesterol (at concentrations of 3.7 mmol/L) and 2.6% (at 5.3 mmol/L) respectively. No women used lipid-lowering agents at any visit.

### Exposure variable – ethnicity

Ethnicity may be defined as the social group a person belongs to, implying a shared culture, history, geographical origin, language, lifestyle factors, physical, genetic and other factors [38]. In this study, ethnic origin was based on the participant’s country of birth or that of the participant’s mother if the mother was born outside Europe or North-America, and further categorized as Western Europe (primarily Norway, Sweden and Denmark), South Asia (primarily Pakistan and Sri Lanka), the Middle East (primarily Iraq, Turkey, Morocco and Afghanistan), Africa (primarily Somalia, Eritrea and Ethiopia), East Asia (primarily Vietnam, Philippines and Thailand), and Eastern Europe (primarily Poland, Russia and Kosovo).

### Covariates

Age of study participants and timing of lipid measurements (GA and weeks postpartum) were used as continuous variables. Gestational age was derived from the first day of the woman’s last menstrual period (LMP), unless LMP date was unknown/uncertain, LMP derived term differed > 14 days from ultrasound derived term (from week 18–20 routine scan) or the pregnancy was a result of in vitro fertilization. Ultrasound term was used in these cases (7% of women). Weeks postpartum at visit 3 was calculated based on offspring`s data of birth. GA/week postpartum was mean centred at each visit. Educational levels were categorised as “primary school or less”, “high school/secondary school” or “college/university” (completed education equivalent to at least a bachelor’s degree). Body height (cm) was measured to the nearest 0.1 cm with a fixed stadiometer (checked against a standard meter before study start and twice yearly). Pre-pregnancy body weight (kg) was self-reported at GA 15, but measured together with total fat mass (Tanita-weight BC 418 MA (Tanita, Tokyo, Japan) at all visits. There was a strong correlation (0.96) between self-reported pre-pregnancy BMI and BMI at GA 15, but some ethnic differences in weight gain before the first visit was found [39].

From the cohort, we have information about a large variety of factors that may be on the causal pathway between ethnicity and lipid levels (i.e. mediators). However, we considered maternal body fat to be the most important possible mediating variable to be included in supplementary analyses, and hence fat mass index (kg/m^2^) was calculated (total fat mass (kg)/height (meter)^2^). Breastfeeding was classified as “exclusive breastfeeding”, “partly” (mixed breastfeeding/formula) and “never”, during the past 14 days prior to the postpartum visit. [40].

### Study sample

In total, 823 women were included at mean GA 15. We excluded women with South- or Central American origin (*n* = 12) due to low numbers, and women with missing values for fasting lipids (*n* = 5) at enrolment, leaving an eligible sample of 806 women with valid data from GA 15 on lipids and lipoproteins. Of these, 759 (94%) women attended at GA 28 and 653 (81%) women attended 14 weeks postpartum (Figure S1). At the postpartum visit, due to resource limitations (including sick-leave among study staff at one study site), women with ethnic minority background were prioritized for fasting blood samples, so we lack data on lipids for about seventy women, primarily ethnic Norwegians [41].

### Statistical analyses

Characteristics of the cohort by ethnic groups are presented by mean values, standard deviation (SD) and numbers/proportions (%). Shapiro–Wilk and the Kolmogorov Smirnov tests indicated that all outcome variables, except triglycerides, were normally distributed. However, we ran models without transforming the triglyceride data, as the linear regression is quite robust to diversion from normality. We performed a rather high number of statistical tests in the current study. To reduce the multiple testing problems, we controlled conservatively the type I error using the Bonferroni correction and set the significance cut-off at α^*^ = α/m = 0.05/ 12 = 0.004, where α = 0.05 and m = 12 is the number of hypotheses generated.

As our aim was to explore the total effect of ethnicity on the concentrations of lipids and lipoproteins, a direct acyclic graph (DAG) was drawn prior to analyses to depict causal structures of possible pathways and associations between ethnicity and the outcomes (Figure S2). Per definition there are no real confounders to these relationships, as no other factors could affect the participant’s ethnicity. However, study inclusion varied by ethnic groups, this could happen as a result of selection. GA (visit 1 and 2), and weeks postpartum (visit 3), age and educational level may be related to selection and to our outcomes, thus we illustrated the selection mechanism in the DAG by including a binary variable S (1 = included, 0 = not included). Several arrows collide in the selection variable S. Study participants by definition have S = 1, this condition induces “collider stratification bias”, which is one type of selection bias [42]. To control for selection bias in the primary analyses exploring the total effect of ethnicity, we adjusted for these covariates associated with selection, but we did not adjust for variables that are part of the causal chain.

To examine ethnic group differences at each of the three time points, we ran linear regression models adjusting for GA or weeks postpartum (Model 1), additionally adjusted for maternal age (Model 2), and additionally adjusted for educational level (Model 3).

We checked the effect of including an interaction term between ethnicity and GA for the four outcomes at each visit, but this did not change the estimates of the ethnic differences (ethnic minority groups compared with Western). We compared the original models with models including interaction terms using the Bayesian information criterion (BIC) (models with lower BIC are generally preferred). The BIC estimate of the models without interaction terms were smaller than the models with interaction terms. Thus, we present the more parsimonious models not including the interaction term.

Linear mixed effect regression models were fitted with an interaction term between time and ethnic group to explore ethnic differences in changes in the outcomes from GA 15 to GA 28 and from GA 15 to 14 week’s postpartum, using similar model building as the cross-sectional analyses.

In Model 4, we also assessed the effect of ethnicity after having closed the mediating path through fat mass by including the simultaneously measured fat mass index (total kg fat mass/m^2^) in the cross-sectional analyses (Model 4). Lastly, we also included maternal breastfeeding, considered as an important mediator, in the analyses of postpartum outcomes (Model 5).

Results from the regression analyses are presented as adjusted means and regression coefficients (β) with 95% confidence intervals (CIs). For consistency when reporting ethnic differences, we used women with Western European origin as reference group. Stata/SE 16.1 was used for all analyses. RStudio version 3.3.2 (2016–10-31) was used to create the figures.

## Results

Ethnic minority women were younger, had lower level of education and were more often multiparous compared with women of Western European origin (Table 1). Ethnic differences were also observed for pre-pregnant BMI and fat mass index at inclusion.

**Table 1:** Characteristics of the cohort by ethnic groups. Values in mean and standard deviation (SD), numbers and percent (%)

	**Total**			**Western Europe**			**South Asia**			**Middle East**			**Africa**			**East Asia**			**Eastern Europe**		
	*n* = 806 (100)			*n* = 333 (41)			*n* = 200 (25)			*n* = 126 (16)			*n* = 60 (8)			*n* = 44 (5)			*n* = 43 (5)		
	n	mean	SD	n	mean	SD	n	mean	SD	n	mean	SD	n	mean	SD	n	mean	SD	n	mean	SD
Gestational age/weeks postpartum																					
Visit 11^a^	806	15.0	(3.4)	333	14.2	(2.3)	200	15.6	(3.9)	126	15.0	(3.3)	60	17.3	(4.9)	44	16.2	(3.9)	43	14.5	(3.0)
Visit 2^b^	759	28.3	(1.3)	315	28.2	(1.2)	192	28.2	(1.2)	117	28.5	(1.4)	52	28.3	(1.3)	40	28.2	(1.1)	42	28.4	(1.7)
Visit 3^c^	652	14.2	(2.8)	279	14.2	(2.7)	163	14.3	(2.9)	99	14.3	(3.0)	39	14.3	(2.5)	36	14.7	(3.2)	36	13.8	(2.3)
Age at enrollment	806	29.8	(4.8)	333	30.9	(4.5)	200	28.6	(4.5)	126	29.5	(5.5)	60	28.3	(5.1)	44	31.2	(4.8)	43	28.9	(4.2)
Pre-pregnant body mass index (kg/m2)	794	24.5	(4.8)	329	24.6	(4.8)	196	23.7	(4.2)	125	25.9	(5.1)	59	25.8	(5.9)	44	22.6	(3.8)	41	23.7	(4.3)
Total body fat (kg) at Visit 1^a^	804	23.5	(9.9)	332	24.1	(9.8)	199	21.6	(8.1)	126	25.3	(10.9)	60	28.5	(12.2)	44	17.8	(8.6)	43	21.8	(7.9)
Fat mass index (kg/m2) at Visit 1^a^	804	8.8	(3.6)	332	8.6	(3.5)	199	8.4	(3.0)	126	9.7	(4.0)	60	10.7	(4.4)	44	7.1	(3.2)	43	7.9	(3.0)
	n	(%)		n	(%)		n	(%)		n	(%)		n	(%)		n	(%)		n	(%)	
Parity	806																				
Nulliparous	373	(46.3)		176	(52.4)		84	(42.0)		44	(34.9)		25	(41.7)		18	(40.9)		28	(65.1)	
Multiparous	433	(53.7)		160	(47.6)		116	(58.0)		82	(65.1)		35	(58.3)		26	(59.1)		15	(34.9)	
Education (years)	800																				
Primary or less	132	(16.5)		10	(3.0)		35	(17.6)		46	(37.1)		28	(46.7)		8	(18.2)		5	(11.9)	
High school/secondary	316	(39.5)		102	(30.8)		101	(50.8)		55	(44.3)		24	(40.0)		19	(43.2)		15	(35.7)	
College/university	352	(44.0)		219	(66.2)		63	(31.7)		23	(18.6)		8	(13.3)		17	(38.6)		22	(52.4)	
Immigrant generation																					
Immigrant	458	(56.8)		36	(10.8)		159	(79.5)		119	(94.4)		59	(98.3)		43	(97.7)		42	(97.7)	
Norwegian-born with immigrant parents	52	(6.5)		1	(0.3)		41	(20.5)		7	(5.6)		1	(1.7)		1	(2.3)		1	(2.3)	
Breastfeeding at Visit 3^c^	631																				
Exclusive	380	(60.2)		173	(64.8)		82	(51.2)		54	(58.1)		23	(59.0)		23	(63.9)		25	(69.4)	
Partial	172	(27.3)		61	(22.6)		55	(34.4)		29	(31.2)		16	(41.0)		7	(19.4)		4	(11.1)	
No breastfeeding	79	(12.5)		33	(12.4)		23	(14.4)		10	(10.7)		0	(0)		6	(16.7)		7	(19.5)	

^a^Visit 1: gestational age 15

^b^Visit 2: gestational age 28

^c^Visit 3: 14 weeks postpartum

Nearly all women with non-Norwegian background were immigrants (born in their country of origin), except for women of Pakistani origin (20.5% born in Norway with immigrant parents) (Table 1). Concentration of lipids and lipoproteins for each visit adjusted for GA/weeks postpartum are shown in Table 2 (and unadjusted values in Supplementary Table 1).

**Table 2:** Concentrations of lipids and lipoproteins (mmol/L) at three time points. Values are numbers, mean and 95% confidence interval (CI)

	**Total**			**Western Europe**		**South Asia**		**Middle East**		**Africa**		**East Asia**		**Eastern Europe**	
	n	Mean	(95%CI)	Mean	(95%CI)	Mean	(95%CI)	Mean	(95%CI)	Mean	(95%CI)	Mean	(95%CI)	Mean	(95%CI)
**Triglycerides (mmol/L)**															
Visit 1^a^	806	1.31	(1.28, 1.35)	1.26	(1.20, 1.31)	1.41*	(1.33, 1.48)	1.37	(1.28, 1.46)	1.04*	(0.90, 1.17)	1.62*	(1.46, 1.77)	1.23	(1.08, 1.39)
Visit 2^b^	750	1.98	(1.93, 2.03)	1.98	(1.90, 2.05)	2.03	(1.93, 2.13)	1.98	(1.86, 2.11)	1.58*	(1.39, 1.77)	2.25	(2.04, 2.46)	1.99	(1.79, 2.20)
Visit 3^c^	576	0.99	(0.95, 1.03)	0.92	(0.86, 0.99)	1.10*	(1.02, 1.18)	1.02	(0.92, 1.12)	0.74	(0.58, 0.89)	1.16*	(0.99, 1.32)	0.93	(0.77, 1.09)
**HDL-cholesterol (mmol/L)**															
Visit 1^a^	806	1.73	(1.70, 1.75)	1.76	(1.72, 1.80)	1.65*	(1.60, 1.70)	1.68	(1.62, 1.74)	1.73	(1.63, 1.83)	1.81	(1.70, 1.92)	1.83	(1.72, 1.94)
Visit 2^b^	750	1.92	(1.89, 1.95)	1.93	(1.88, 1.98)	1.87	(1.81, 1.93)	1.89	(1.81, 1.97)	1.88	(1.76, 2.00)	2.12	(1.98, 2.26)	1.97	(1.84, 2.11)
Visit 3^c^	576	1.54	(1.51, 1.57)	1.60	(1.55, 1.65)	1.43*	(1.37, 1.49)	1.54	(1.47, 1.62)	1.67	(1.55, 1.79)	1.51	(1.39, 1.64)	1.52	(1.39, 1.64)
**LDL-cholesterol (mmol/L)**															
Visit 1^a^	802	2.75	(2.69, 2.80)	2.74	(2.66, 2.83)	2.78	(2.67, 2.88)	2.75	(2.62, 2.88)	2.81	(2.62, 3.00)	2.43	(2.20, 2.65)	2.83	(2.61, 3.06)
Visit 2^b^	739	3.45	(3.38, 3.53)	3.62	(3.51, 3.74)	3.31*	(3.17, 3.45)	3.28*	(3.10, 3.46)	3.40	(3.14, 3.67)	3.03*	(2.72, 3.34)	3.78	(3.48, 4.07)
Visit 3^c^	575	3.07	(3.01, 3.14)	3.09	(2.98, 3.20)	3.13	(3.00, 3.26)	2.98	(2.82, 3.15)	2.95	(2.69, 3.21)	3.04	(2.75, 3.32)	3.10	(2.83, 3.38)
**Total cholesterol (mmol/L)**															
Visit 1^a^	806	5.00	(4.94, 5.05)	5.01	(4.92, 5.11)	4.99	(4.87, 5.11)	4.97	(4.82, 5.12)	4.96	(4.74, 5.18)	4.89	(4.64, 5.14)	5.12	(4.87, 5.38)
Visit 2^b^	750	6.18	(6.10, 6.26)	6.36	(6.24, 6.48)	6.02*	(5.86, 6.17)	5.93*	(5.73, 6.14)	5.92*	(5.62, 6.23)	6.14	(5.80, 6.48)	6.57	(6.24, 6.91)
Visit 3^c^	576	5.02	(4.95, 5.10)	5.08	(4.96, 5.20)	5.01	(4.87, 5.15)	4.95	(4.77, 5.13)	4.92	(4.63, 5.21)	5.04	(4.73, 5.34)	5.01	(4.70, 5.31)

^a^Visit 1: gestational age 15

^b^Visit 2: gestational age 28

^c^Visit 3: 14 weeks postpartum

Adjustments for gestational age/weeks postpartum

Ethnic groups that differed statistical significant (*p* < 0.004) from the Western European group (reference) are marked with*

Statistics: Linear regression

### Lipids and lipoproteins s and changes during pregnancy to postpartum

**Fig. 1:**
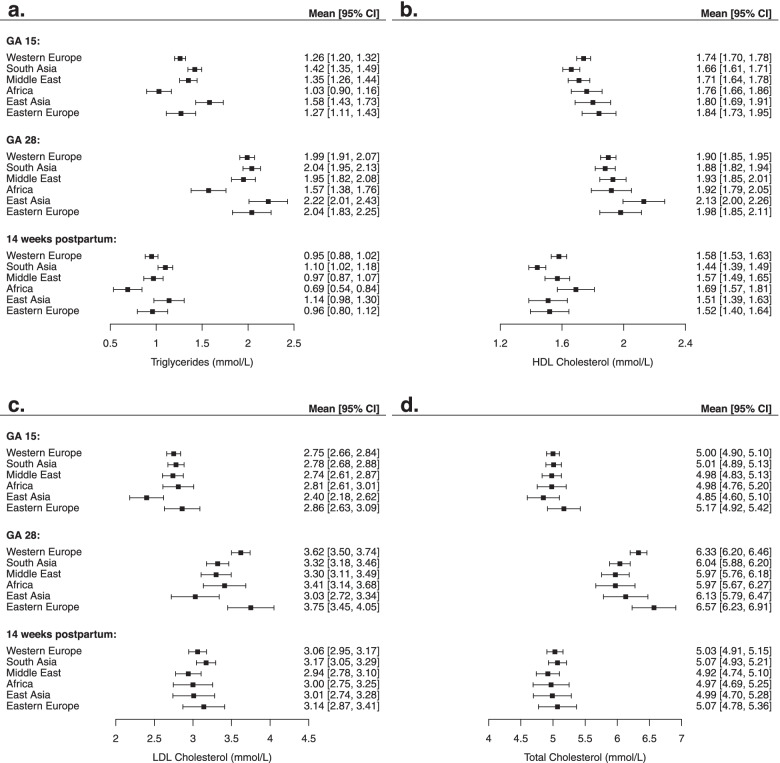
**a** Mean (95% CI) triglycerides (mmol/L) at gestational age 15, 28 and 14 weeks postpartum among ethnic groups. **b** Mean (95% CI) HDL-cholesterol (mmol/L) at gestational age 15, 28 and 14 weeks postpartum among ethnic groups. **c** Mean (95% CI) LDL-cholesterol (mmol/L) at gestational age 15, 28 and 14 weeks postpartum among ethnic groups. **d** Mean (95% CI) cholesterol (mmol/L) at gestational age 15, 28 and 14 weeks postpartum among ethnic groups. Adjusted for gestational age, week postpartum, age and education. Western Europe *n* = 333, South Asia *n* = 200, Middle East *n* = 126, Africa *n* = 60, East Asia *n* = 44, Eastern Europe *n* = 43

Estimates obtained from the cross-sectional analyses (Model 1) changed marginally after additional adjustments for maternal age (Model 2) and education (Model 3) (Table S1a-c). Results for Model 3 are visualized in Fig. 1a-d. Compared with women of Western European origin, triglycerides were lower in women with African origin at GA 15, and higher in women of South- and East Asian origin. For HDL, LDL- and total cholesterol no ethnic differences were observed (Fig. 1a-d).

**Fig. 2:**
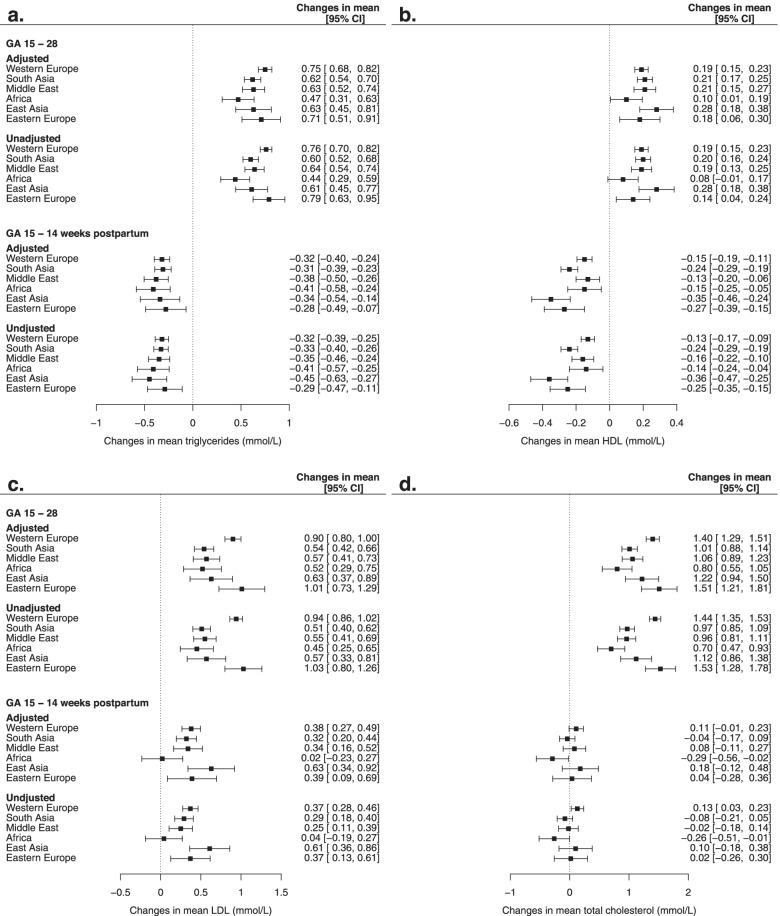
**a** Changes in triglycerides (mmol/L) from gestational age 15 to 28 and from gestational week 15 to 14 weeks postpartum by ethnic groups. **b** Changes in HDL- cholesterol (mmol/L) from gestational age 15 to 28 and from gestational week 15 to 14 weeks postpartum by ethnic groups. **c** Changes in LDL-cholesterol (mmol/L) from gestational age 15 to 28 and from gestational week 15 to 14 weeks postpartum by ethnic groups. **d** Changes in total cholesterol (mmol/L) from gestational age 15 to 28 and from gestational week 15 to 14 weeks postpartum by ethnic groups. Adjusted for gestational age, week postpartum, age and education. Values in β and 95% CI

Changes in the concentrations of lipids and lipoproteins (during pregnancy and from GA 15 to 14 weeks postpartum) are presented in Tables S2a-b and Fig. 2a-d. From GA 15 to 28 all lipids and lipoproteins increased in all ethnic groups. In women of Western European origin, triglycerides increased by 60%, LDL-cholesterol by 32%, total cholesterol by 26% and HDL-cholesterol by 9% (Fig. 2a-d). The increase in triglycerides was smaller in women of African and South Asian origin, and the increase in LDL- and total cholesterol was generally smaller in women of non-European origin compared with Western and Eastern Europeans. Therefore, at GA 28, compared with women of Western European origin, triglycerides were lower in women with African origin. LDL-cholesterol lower in women of South Asian and East Asian origin. No ethnic differences was observed for total cholesterol. The concentration of HDL-cholesterol was higher in women with East Asian origin (Fig. 1a-d).

At 14 weeks postpartum, the concentrations of triglyceride and HDL-cholesterol were reduced compared with GA 15 in all ethnic groups, while concentrations of LDL-cholesterol were higher in all groups, and no changes were observed for total cholesterol (except for women of African origin) (Fig. 2a-d). The reduction in HDL-cholesterol was more pronounced in women of South- and East Asian origin and Eastern Europeans compared with Western Europeans. At the postpartum visit, triglycerides were lower in women of African origin compared with Western Europeans. Women of South Asian origin had lower HDL-cholesterol, while no ethnic differences were observed for LDL-and total cholesterol (Fig. 1a-d).

We also explored the direct effect of ethnicity after additionally adjusting for fat mass index (kg/m^2^) (Model 4 in Tables S1a-c, S2a-b), and found that all estimates for lipids and lipoproteins at each time-point and for their changes during pregnancy and from GA 15 to 14 weeks postpartum changed only marginally. Further, adding breastfeeding to the model at the postpartum visit had no impact on the effect estimates for the ethnic differences (Model 5 in Tables S1c).

## Discussion

### Main findings

To the best of our knowledge, this is the first study to report ethnic differences in the concentrations of lipids and lipoproteins during pregnancy to postpartum in a multi-ethnic population. Ethnic differences varied by GA and type of lipids and lipoproteins. Compared with Western Europeans, women of African origin had lower triglycerides at all time-points, while women with origin from South Asia had higher triglycerides at GA 15, and lower HDL-cholesterol postpartum. As the increase in triglycerides, LDL- and total cholesterol during pregnancy was generally smaller in most non-European ethnic minority groups, Europeans had the highest LDL- and total cholesterol at GA 28, while East Asians had the lowest LDL- and the highest HDL-cholesterol. At the postpartum visit, triglycerides and HDL-cholesterol concentrations were lower than in GA 15, but the mean concentration of LDL-cholesterol were still higher than in early pregnancy in all groups except for Africans.

### Strengths and limitations

The strengths of this study include the population-based design, the large proportion of ethnic minority women (mostly immigrant women), minor loss to follow-up [35] and measurements of fasting lipids measured by standard methods from three time-points. When we explored the direct effect of ethnicity on the lipids and lipoproteins, we used fat mass index, as this measure may be more relevant than BMI when investigating the mediating effect of bod fat. However, as ethnic differences in gestational weight gain were present, this may have introduced some bias when adjusting for fat mass index. Further, heterogeneity within relatively broad ethnic groups probably exists and the number in some ethnic groups was low, and information about concentrations of lipids and lipoproteins before pregnancy is lacking.

### Interpretation

Through our systematic search we identified only five studies assessing ethnic differences in lipids and lipoproteins in pregnancy; three from Europe [31–33] and two from the US [14, 43], all based on one single measurement, and none reported changes during pregnancy or from pregnancy to postpartum. Comparisons are further hampered by methodological issues such as differences in design (prospective, case control and cross-sectional studies), timing of measurements, fasting status, differential adjustment for confounders and mediators, and different ethnic groups included. Some studies included mainly high-risk groups [14, 32], thus not representative for the general population of pregnant women. One study from UK found that women of African origin had lower fasting triglycerides, LDL- and total cholesterol and higher HDL-cholesterol at GA 30 than Caucasians [32], in line with our results from GA 28 (for triglycerides, LDL-and total cholesterol), with studies from the US [14, 43] and with studies outside pregnancy [30]. Similarly, lower non-fasting total cholesterol was found in early pregnancy in African-Caribbean, Ghanaian and Moroccan women (in contrast to our findings), and lower triglycerides in Ghanaians than in Dutch women, while Turkish and South Asian origin (Surinam-Hindustani) had slightly higher levels, in line with our findings [33]. Two other studies have also found that women of South Asian origin had higher concentrations of triglycerides than Europeans both in early pregnancy [43] and at GA 26 [31].

Pregnancy is considered a “natural stress test” for women, as complications like GDM and preeclampsia seem to be early markers of metabolic disturbances, endothelial dysfunction and/or hypertension that predict future risk of type 2 diabetes [26], and CVD [44].

It was beyond the scope of this study to assess the very complex interplay between dysglycaemia and dyslipidaemia and the possible relations with pregnancy complications, such preeclampsia and GDM. We have previously shown that women of South Asian origin have a higher risk of GDM and insulin resistance during pregnancy and postpartum [45], higher postpartum weight retention [46] and fat mass [47]. This is in line with studies outside pregnancy [29] and in childhood [48]. The earlier onset of type 2 diabetes and CVD in South Asians than in Europeans [49, 50], seems to be partly related to differences in body composition and a particular susceptibility for an obeso-genetic environment [29]. The healthier lipid profile outside pregnancy for African origin populations [30], which may be reflected in our study, seems to be related to differences in physiology, with relatively more accumulation of fat in the subcutaneous than visceral compartment, greater lipoprotein lipase activity and a higher insulin response than in Europeans [30]. The risk of CVD in subjects of African origin seems to be more driven by blood pressure, while associations with lipids and lipoproteins are weaker [30, 51].

The pregnancy induced elevations in lipid concentrations usually drop within 24 h postpartum[4], while LDL-cholesterol may remain elevated for at least seven weeks postpartum [3]. We found that levels of LDL-cholesterol were still higher than at GA 15 at the visit 14 weeks postpartum in all ethnic groups, except in women of African origin. If we assume that the ethnic differences in early pregnancy reflect similar differences before conception, pregnancy might promote an adverse development in LDL-cholesterol and risk of CVD, in line with what we previously have found for development of blood pressure [52] and postpartum weight retention [46], and is worrisome in relation to the next pregnancy [53].

Socioeconomic status, contextual and cultural factors, and the degree of social integration in the country of residence, impose a strong influence on lifestyle of ethnic groups who have left their country of origin, not least the diet [54], over generations [29]. Therefore, we cannot infer that our results among immigrant women are reflected in those of pregnant women in their country of origin. We treated Eastern European women as a separate group despite small numbers, because their phenotype and their concentrations of lipids and lipoproteins differed from Western European women (the reference group). Although socioeconomic differences between these two regions have declined over the last decades, pregnant women born in Eastern Europe have most likely been exposed to more poverty and other norms regarding lifestyle factors than Western European women. Although we adjusted for education, differences in the diet could possibly have contributed to the ethnic differences observed, mediated through body fat [30] or weight gain during pregnancy [55]. Our secondary analyses did however indicate that maternal body fat contributed only marginally to the observed ethnic differences in lipids and lipoproteins. Body composition, the proportion of visceral fat, the role of lipoprotein lipase and of insulin resistance differ by ethnicity, probably related to genetics and/or epigenetics [28–30], and influence lipid and lipoprotein metabolism. Furthermore, hormonal changes (e.g. oestrogen) may drive the increase in triglycerides [7], but little is known about ethnic differences in hormone levels during pregnancy and postpartum. Human breast milk has a high content of triglycerides, and women who breastfeed tend to have lower concentrations of total cholesterol, triglycerides and very low density lipoprotein (VLDL)-cholesterol than women who do not breastfeed [8]. Pregnancy induces an atherogenic lipid profile, which seems to be partly reversed by lactation [8, 56]. Nevertheless, ethnic differences in lipid concentrations postpartum were not explained by differences in breastfeeding in our study.

The most consistent associations between lipid concentrations and foetal growth and other pregnancy outcomes seem to be present for triglycerides [2]. We only identified two studies exploring relations between lipids and pregnancy outcomes like preterm delivery [14], pregnancy-induced hypertension, preeclampsia and foetal growth in multi-ethnic samples, with similar results before and after adjustments for ethnicity [34]. However, one study assessing the relation between maternal lipid genetic risk scores and foetal growth, found that associations for triglyceride scores varied by ethnicity, obesity status and offspring sex [43]. From our cohort, we have previously reported that birth weight was lowest in offspring of mothers of Asian origin [57, 58], the ethnic group with the highest triglyceride levels during pregnancy in this study. Further, HDL-cholesterol at GA 28 was inversely associated with birth weight, but not with neonatal sum of skinfolds, and no strong associations with triglycerides were observed [22]. However, LDL-cholesterol is also an important source of cholesterol for the foetus, and the syncytiotrophoblast, the functional unit of the placenta, can take up maternal LDL-cholesterol particles by endocytosis [59]. At this stage we can only speculate if the lower LDL-cholesterol at GA 15 and the smaller increase in South- and East Asians during pregnancy may be related to slower foetal growth and smaller offspring size [24, 60]. If so, ethnic differences in placental transfer and metabolism of lipids and lipoproteins may be present, probably involving complex mechanisms [10].

In conclusion, increased awareness among clinicians about the striking differences in concentrations of lipids and lipoproteins observed during pregnancy to postpartum between several ethnic groups living in the same the country seems indicated. A better understanding of causes for these observed ethnic differences in lipids and whether they can be linked to ethnic differences in pregnancy outcomes and long term effects for women and their children are needed. Further, larger studies are recommended to study whether these effects are similar across ethnic groups.

## Supplementary Information

**Additional file 1:** **Figure S1.** Flow chart of study sample selection. Percentages for "did not attend" and "missing" are based on total number of participant (*n*=811) at visit 1 and by ethnic group.

**Additional file 2:** **Figure S2.** Causal diagram for the association between ethnicity and plasma lipid. S is defined as a binary selection variable. Our study participants have S=1. U = women included at different time points from early pregnancy to 14 weeks postpartum. The total effect of ethnicity on plasma lipids is found by adjusting for gestational week, education and age.

**Additional file 3:** **Table S1a.** Concentrations of lipids and lipoproteins (mmol/L) at gestational age (GA) 15 by ethnic groups. Values in mean and 95% CI. **Table S1b.** Concentrations of lipids and lipoproteins (mmol/L) at gestational age (GA) 28 by ethnic groups. Values in mean and 95% CI. **Table S1c.** Concentrations of lipids and lipoproteins (mmol/L) at 14 weeks postpartum by ethnic groups. Values in mean and 95% CI. **Table S2a.** Changes in concentrations of lipids and lipoproteins (mmol/L) from gestational age (GA) 15 to 28 by ethnic groups. Values in β and 95% CI. **Table S2b.** Changes in concentrations of lipids and lipoproteins (mmol/L) from gestational age (GA) 15 to 14 weeks postpartum by ethnic groups. Values in β and 95% CI.

## Data Availability

The editors can access data (in de-identified form) used in the manuscript, code book, and analytical code upon request. The project manager and the head of our department will contribute to the access being provided under appropriate conditions. However, research data for this publication include identifying health information subject to confidentiality. It is therefore not possible to share raw data.

## Abbreviations

HDL-cholesterol: High-density lipoprotein cholesterol
LDL-cholesterol: Low-density lipoprotein cholesterol
GDM: Gestational diabetes
CVD: Cardiovascular disease
GA: Gestational age
DAG: Direct acyclic graph
