# Supplementary figures and images for: Lipid and lipoprotein concentrations during pregnancy and associations with ethnicity
Source: BMC Pregnancy Childbirth. 2022 Mar 24;22:246. doi: 10.1186/s12884-022-04524-2 (PMC8953044; doi:10.1186/s12884-022-04524-2)

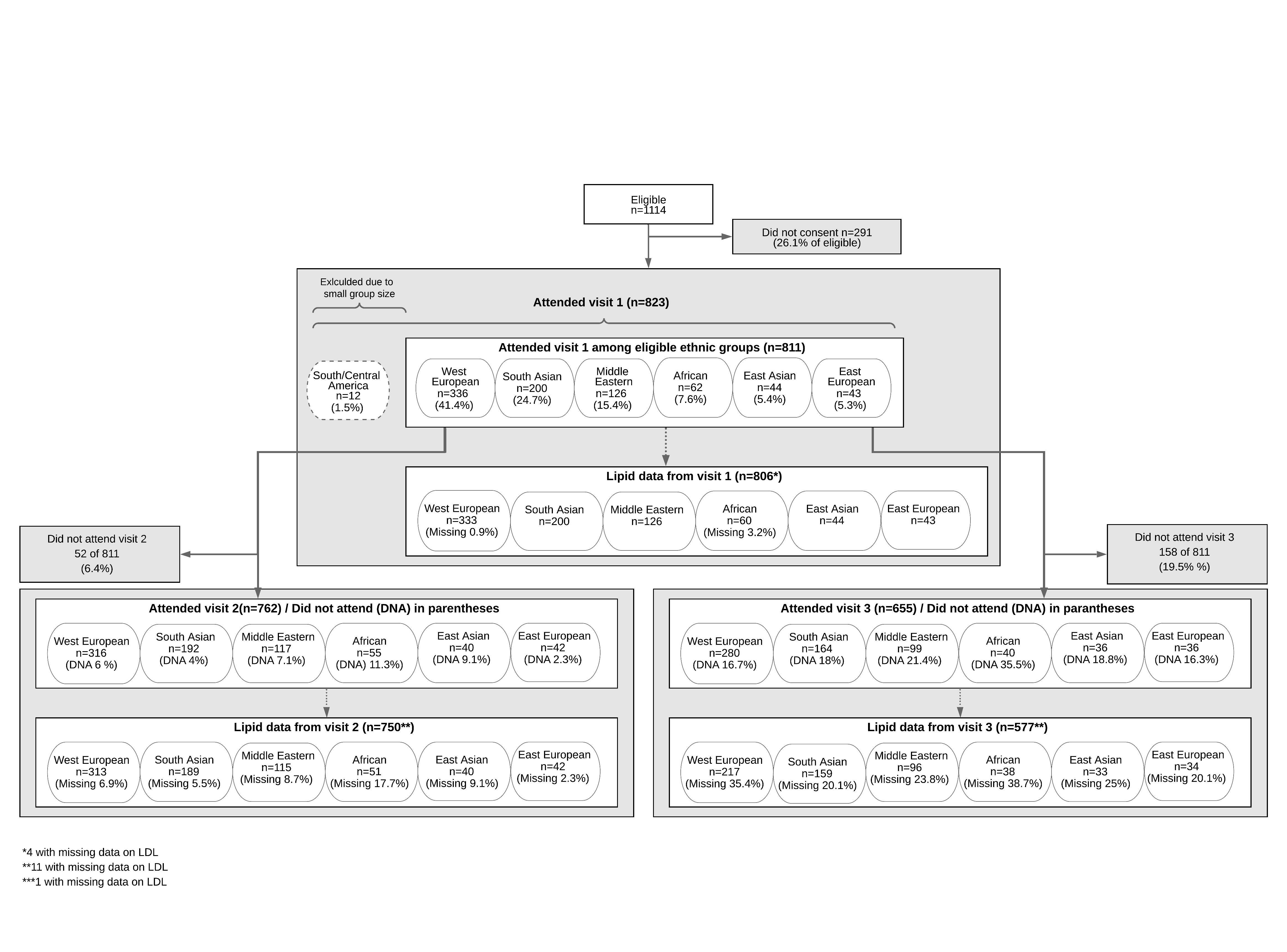

Supplement: Supplementary file 1 — Additional file 1: Figure S1. Flow chart of study sample selection. Percentages for "did not attend" and "missing" are based on total number of participant (n=811) at visit 1 and by ethnic group. [file 12884_2022_4524_MOESM1_ESM.jpeg]
