# Supplementary material for: Lipid and lipoprotein concentrations during pregnancy and associations with ethnicity
Source: BMC Pregnancy Childbirth. 2022 Mar 24;22:246. doi: 10.1186/s12884-022-04524-2 (PMC8953044; doi:10.1186/s12884-022-04524-2)
Supplement: Supplementary file 2 — Additional file 2: Figure S2. Causal diagram for the association between ethnicity and plasma lipid. S is defined as a binary selection variable. Our study participants have S=1. U = women included at different time points from early pregnancy to 14 weeks postpartum. The total effect of ethnicity on plasma lipids is found by adjusting for gestational week, education and age. [file 12884_2022_4524_MOESM2_ESM.docx]

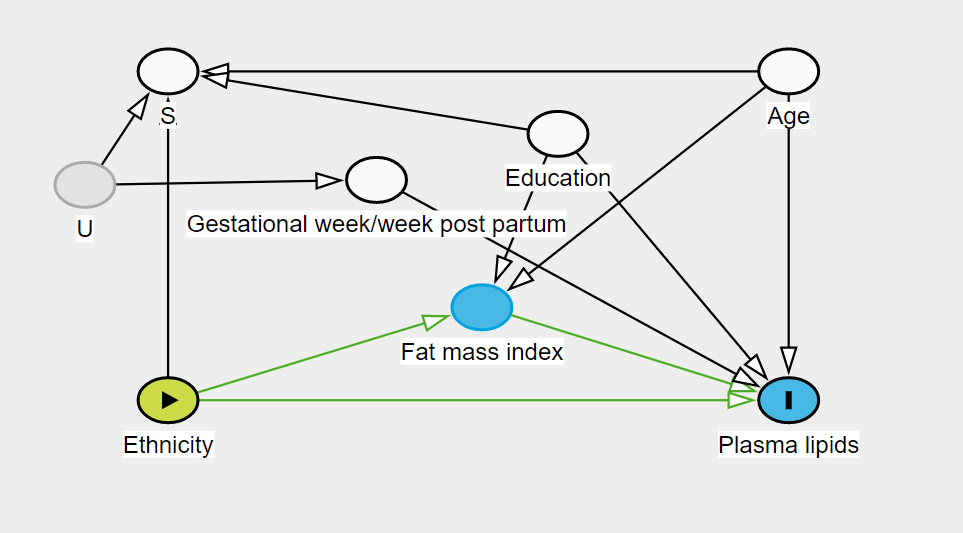


**Figure S2.** Causal diagram for the association between ethnicity and plasma lipid. S is defined as a binary selection variable. Our study participants have S=1. U = women included at different time points from early pregnancy to 14 weeks postpartum. The total effect of ethnicity on plasma lipids is found by adjusting for gestational week, education and age.
